# The Effect of HBV/HCV in Response to HAART in HIV Patients after 12 Months in Kumba Health District in the South West Region of Cameroon

**DOI:** 10.3390/tropicalmed6030150

**Published:** 2021-08-10

**Authors:** Adamu Ndongho Ndifontiayong, Innocent Mbulli Ali, Jean Baptiste Sokoudjou, Jerimiah Mbogwe Ndimumeh, Christopher Bonglavnyuy Tume

**Affiliations:** 1 Research Unit of Microbiology and Antimicrobial Substances, Faculty of Science, University of Dschang, Dschang P.O. Box 67, Cameroon; adamndifontiayong@yahoo.com (A.N.N.); dr.alinn@gmail.com (I.M.A.); sokoudjouwls@yahoo.fr (J.B.S.); ndimumehjerimiah@gmail.com (J.M.N.); 2 Laboratory for Public Health Research Biotechnologies, The Biotechnology Centre, University of Yaoundé 1, Yaoundé P.O. Box 8094, Cameroon; 3 Département des Sciences Appliquées à la Santé, Institut Universitaire et Stratégique de l’Estuaire (IUEs/Insam), Douala BP 4100, Cameroon; 4 Department of Biochemistry, Faculty of Science, University of Bamenda, Bamenda 00237, Cameroon

**Keywords:** HIV, hepatitis B and C, co-infection, immune response, hepatotoxicity, Kumba

## Abstract

Hepatitis B (HBV) and C (HCV) are two among the numerous forms of infections whose clinical degeneration, morbidity–mortality and low immune responsiveness in people living with human immunodeficiency virus (HIV) are highly evident. Co-infection of HIV with HBV and HCV has been associated with reduced survival, increased risk of progression to liver diseases and increased risk of hepatotoxicity associated with antiretroviral therapy (ARV). We carried out biochemical, immunological, virological and clinical analysis of hepatitis B and C positive HIV patients as well as some HIV positive individuals receiving antiretroviral therapy in Kumba Health District to evaluate the immune response to the ARV therapy and identified risk factors associated with the treatment outcomes. A total of 52 HIV patients, 36 HIV/HBV and 12 HIV/HCV patients were involved in this study. We performed CD4 counts, viral load test, analyzed ALAT/ASAT, albumin, bilirubin, and creatinine and measured the weights of HIV patients, HIV/HBV and HIV/HCV enrolled for not more than one year in Kumba Health District. The results were analyzed to evaluate the immune response and possible risk factors associated with the treatment outcomes. The mean increase in weight in participants of all groups over 12 months (17.12 kg) was greater than the mean increase in CD4 (8.92 cell/mm^3^). However, the mean decrease in viral loads over a 12 months was also very high (1035.17 copies/mL). There was a significant change in the mean values from baseline for all the three variables (*p* < 0.0001). HIV disease outcomes following HAART (high active antiretroviral therapy) do not appear to be adversely affected by HBV or HCV co-infection, except for slightly poorer CD4 count responses in HIV/HCV co-infected patients. Concerning the renal and liver functions, all the biomarkers witnessed a decrease in patients of all groups in response to HAART over time, with a more rapid decrease in mono-infected patients as compared with those co-infected with HBV but the case was contrary for those co-infected with HCV. Co-infection with HBV or HCV was relatively common among HIV infected participants in Kumba Health District. There were differences in response to HAART between the mono-infected compared with the co-infected, taking into consideration the weight, CD4 count, and viral load. In addition, there was also a variation in the different biomarkers of liver and renal function between mono-infected and co-infected patients.

## 1. Introduction

Viral hepatitis has emerged as an important public health problem globally. It is characterized by high prevalence, a high burden in terms of morbidity and mortality, and suboptimal diagnosis and management approaches in developing countries [1,2]. Hepatitis B (HBV) and C (HCV) are two among the numerous forms of infections whose clinical degeneration, morbidity–mortality and poor immune responsiveness in people living with human immunodeficiency virus (HIV) are highly evident [3,4]. Co-infection of HIV with HBV and/or HCV has been associated with reduced survival, increased risk of progression to liver diseases and increased risk of hepatotoxicity associated with antiretroviral therapy [3,4]. Approximately, 550,000 million people (~9% of the world population) are chronically infected with either hepatitis B virus (HBV; 350–400 million) and/or hepatitis C virus (HCV; 170–180 million) worldwide [4,5]. HBV and HCV account for 60% of cirrhosis, 80% of hepatocellular carcinoma and 1 million deaths each year and the burden of chronic hepatitis mostly affects resource-limited countries [6].

Studies have shown that the worldwide, the prevalence of HBV ranges from 0.1% to 20% [7,8]. HBV is detected in blood and body fluids (semen, saliva and nasopharyngeal fluid), and the four major modes of transmission are: sexual contact, mother to child transmission in pregnancy and at birth, blood to blood contact and through sharing of infected items [9]. The world’s predominant mode of transmission of HBV is perinatal. In this case, children born of a HBV positive mother have 90% risk of having the infection too, among which 25% will die in adult life of chronic liver disease or cancer [10]. Both HIV and HBV exert reciprocal influence on the disease progression due to the other and the impact in terms of mortality and morbidity is known [11]. The impact of HBV/HIV in low resource settings remains a concern [11,12].

Hepatitis C virus is also detected in blood and body fluids, and transmitted from an infected person to a non-infected person in a manner similar to that of HBV. HIV/HCV co-infection is being recognized as a separate entity from HIV or HCV mono-infections [13]. Further research is thus needed in order to better understand how the natural history of each virus is modified by the presence of the other, and to determine the exact mechanism of liver toxicity in order to maximize the effectiveness of current therapeutic regimens in co-infected patients.

Chronic hepatitis is a common cause of liver-related morbidity due to different hepatic viruses, where hepatitis B (HBV) and hepatitis C (HCV) have been identified as the main cause and lead to many complications. Over 1 million persons die annually due to HBV-related complications and such disease is rare in western countries and acquired primarily at adulthood, whereas in Asia and most Africa countries, chronic HBV infection is common and usually acquired prenatally or in childhood [14]. HCV also leads to many complications including HCC (hepatocellular carcinoma) in 32% of infected patients. Infection with multiple viruses leads to management problems with higher incidence of morbidity and mortality.

Scientists carrying out studies in this perspective have shown that HBV, HCV and HIV are endemic in Africa, especially the sub-Saharan Africa. With increasing access to highly active ARV therapy, the burden as well as the consequences of persistent of HBV/HCV infections among the HIV patients in resource-limited countries is expected to rise as opposed to the situation in Europe and North America [11,12]. Other studies have suggested that HIV/HCV co-infected patients have a blunted immune response to HAART, compared with those with HIV infection alone [15], although others have found comparable degrees of immune restoration in persons with HIV/HCV co-infection [16].

In Cameroon, a meta-analysis showed the seroprevalence of hepatitis B among HIV-1 infected patients was 12.0% and 4.0% for HCV in our previous study [17] in Cameroon. It is of importance to assess the extent of the problem in key health districts with ongoing generalized epidemics of HIV-1 infection. In addition, if not properly addressed, hepatitis-virus-related diseases may threaten to stall the success of ARV therapy in the developing country settings [18]. Guidelines for clinical management of HIV patients recommends screening and characterization for viral hepatitis; unfortunately this is not standard practice in Cameroon, as it is not included in the package of baseline laboratory tests [19]. Given the lack of data from the health district data base, there is a need to determine the scope of viral hepatitis burden, immune response and treatment outcomes among the HIV-positive individuals in the Kumba Health District in order to raise awareness of the importance of this problem. 

We determined the prevalence of HBV/HCV among HIV-1 infected patients in the Kumba Health District [17] and in this article, we assessed changes in key immune, hepatic and renal function biomarkers and studied risk factors associated with treatment outcomes among coinfected (HIV/HBV, HIV/HCV) and HIV-1 monoinfected patients in Kumba Health District over a 12 months follow up period.

## 2. Material and Methods

### 2.1. Study Design

This study was a prospective cohort study involving mono- and hepatitis-co-infected HIV participants followed up over 12 months in the Kumba Health District. 

### 2.2. Study Procedures and Sample Analysis

Participants for this study were selected from 299 participants of a previous cross-sectional hypothesis-driven study [17] to constitute cohorts of co-infected or mono-infected individuals. The previous baseline study details the inclusion and exclusion criteria. Briefly, patients who were free of tuberculosis, at most one year on HAART, at most at stage III of HIV infection and who were not option B+ were included. Anyone at stage IV of HIV infection, pregnant women before the selection, tuberculosis (TB) patients and patients with any other underlying clinical conditions or who has been on treatment for more than 12 months was excluded. Eligible participants in the cohort study were invited for clinical examination during routine HIV appointments, during which samples were collected and analyzed in the laboratories at the study sites. Data were collected from the 52 HIV, 36 (HIV/HBV) and 12 (HIV/HCV) studied participants after sample analysis. For the present study, sample analysis was performed using PIMA Analyzer For CD3+/CD4+ Cell Count; Cypress diagnostics, Lot: GOT-00371A, Ref: HBEL010 for the measurement of serum aspartate amino-transferase (AST); Cypress diagnostics, Lot: GPT-00371A, Ref: HBEL020 for the measurement of alanine amino transferase (ALT); colorimetric Rand and Pasqua, 1982 method for the measurement of total and direct bilirubin; and a UV–VIS spectrophotometer for albumin and creatinine levels. All samples for viral load measurement were centrifuged and plasma transported, according to the routine procedure, to the Baptist Hospital in Mutengene (BHM) located 1 h away from the collection site. At the BHM, the Abbott Real Time (m2000sp) assay was performed to quantify HIV-1 RNA with the reportable range of 40 to 10,000,000 HIV-1 RNA copies per milliliter. Results were validated by a quality assurance officer and returned to the clinical site via a paper and web-based system. We analyzed participant samples for CD4 counts, ALAT/ASAT, albumin and body weight at three monthly intervals while the viral load was estimated every six months. Sample analysis was offered free of charge to all the 100 participants. All the participants in the study were placed HAART (Tenofovir Lamivudine Efavirenz) as required by the national guideline.

Ethical clearance for this study was obtained on 15 November 2018 from the Faculty of Health Science Institutional Review Board, University of Buea following the protocol Reference no: 2018/814-05/UB/SG/IRB/FHS. This study was implemented from 16 November 2018 to 16 November 2019.

### 2.3. Treatment Outcomes of HIV/HBV and HIV/HCV Co-Infected Patients Following HAART

The endpoints for the clinical, virological and immunological response of co-infected and mono-infected patients were a 12-month variation in patient weight, detectable viral load and variations in CD4 cell counts.

### 2.4. Statistical Methods and Data Analysis

Data were entered into Microsoft Excel. The different variables included outcome variables like: age, sex, weight, CD4 count, viral load, ALAT and ASAT enzyme activities, and albumin, bilirubin and creatinine concentrations as well as hepatitis status.

Descriptive statistics were performed for all variables and binary logistic regression was used to determine the possible factors associated with treatment outcomes. The baseline was defined from the date when all the above-mentioned parameters including the parameters for liver and renal function tests were analyzed. The effect of hepatitis co-infection on viremia at 12 months after baseline was assessed by logistic regression among patients with baseline viral load measurement. The effect of hepatitis co-infection on immunological progression was assessed by fitting a linear model on time-weighted average changes in CD4 counts from baseline to 12 months. Changes in ALAT/ASAT, albumin, and bilirubin as well as creatinine levels for renal function were also assessed at baseline, during and at the end of 12 months. Covariates tested for inclusion in all multivariate models were: HIV positive category, HIV stage of at most III, been on HAART for not more than one year, be free of TB, not pregnant and should be on regular therapy in the study sites. Each covariate was tested at the 5% level using the chi-squared test for heterogeneity if it was a nominal variable or a *t*-test for trend if it was ordinal. Statistical analysis was done using the statistical software SPSS version 20 and Epi Info version 7.0.

## 3. Results

### 3.1. Response to Treatment of HIV Patients Co-Infected with HBV and HCV

The following parameters were used to monitor response to treatment of HIV patients and HIV patients co-infected with HBV and HCV are shown in Table 1.

The mean change from baseline was computed for each variable and a one sample *t*-test was used to assess for a significant change from baseline for all the variables.

Table 1 shows that the percentage increase in weight at 95% CI over 12 months (17.12 kg) was greater than the percentage increase in CD4 (8.92 cells/mm^3^). However, the percentage decreased in viral load over a 12-month period was also very high (1035.17 copies/mL). The above data showed that there was a significant change in mean value from baseline for all the three variables.

To assess if there was a significant difference in immune response among the mono-infected and co-infected patients, an independent sample *t*-test was conducted with percentage change as the outcome variable and HBV and HCV status as the explanatory variable.

The results from the group statistic Table 2 above shows that mono-infected patients had an increase in weight (mean = 22.57 kg and 18.65 kg) while co-infected (HBV/HIV) patients had an increase in CD4 (mean = 10.41 cells/mm^3^ and 8.08 cell/mm^3^), and a significant decrease in viral load (mean = 1319.67 copies/mL and 1111.20 copies/mL) for HIV/HBV and HIV, respectively, contrary to HIV/HCV.

The independent samples *t*-test shows that the percentage increase in weight was significantly different in participants co-infected with HBV (*p <* 0.0001) but not with HCV (*p* = 0.064) or all three. Increase in CD4 by HBV or HCV status of HIV-1 patients was not significant (*p* = 0.375 for HBV and *p* = 0.544 for HCV). Same observation was made when comparing percentage decrease in viral load counts between mono-infected and co-infected patients.

Since we observed a difference in weight gain by co-infection status, we decided it was important to further conduct the one-sample *t*-test for the different categories in our sample separately. The results showed that there was a significant change from baseline for all the three variables for both mono-infected HIV and co-infected HIV/HBV and HIV/HCV patients as seen in Table 3 above.

### 3.2. Renal Function of Co-Infected HIV/HBV, HIV/HCV and Mono-Infected HIV Patients

The creatinine percentage change from baseline was computed and one sample *t*-test was used to assess if there was a significant difference between baseline measurement and measurement after 12 months.

The result from the one sample test shows that there is a significant difference between creatinine baseline measurement and measurement after 12 months (*p* = 0.020).

In order to assess if there exists a significant difference in renal function outcome between mono-infected and co-infected HIV/HBV and HIV/HCV patients, an independent sample *t*-test was conducted with percentage change in creatinine levels from baseline as the outcome variable and HBV infection status as the explanatory variable.

Table 4 below shows an increase in percent change in creatinine levels (mean = 11.1111 mg/dL) in HIV/HBV co-infected patients compared with mono-infected patients (mean = 6.2500 mg/dL). On the contrary, the mean percentage change in creatinine levels was higher in HIV/HCV co-infected patients (mean = 9.0909) compared with mono-infected (mean = 0.0000). However, this difference was not statistically significant (*p* = 0.494) at the 0.05 threshold.

With regards to baseline, Table 5 below shows separately the difference in creatinine level from baseline for each of the co-infection status. From this one sample test, there was a significant difference in creatinine level from baseline for HIV/HBV co-infected patients at *p*-value of 0.044 but not for mono-infected patients. On the other hand, *t*-test could not be computed for HIV/HCV co-infected patients because the standard deviation was 0.

### 3.3. Liver Toxicity for HIV and HIV/HBV and HIV/HCV Co-Infections

In order to assess the level of liver toxicity in the study participants, the activity of ALAT/ASAT, levels of serum albumin, and total bilirubin were measured at baseline and at 12 months and comparisons between these two time points were made. The percentage change from baseline was computed for all these variables and a one sample *t*-test was used to establish the overall change from baseline for the variables.

Table 6 below shows the results of this analysis indicating a significant percentage decrease in ALAT (20.03 IU/L) and ASAT (12.78 IU/L) activity over 12 months (20.03 IU/L) from baseline (*p* = 0.001 in each case). There was also a slight decrease in albumin and total bilirubin concentrations from baseline but this change was not statistically significant (*p* = 0.103 and *p* = 0.242, respectively).

In order to assess if there was a change in liver function between mono-infected and co-infected patients, an independent sample *t*-test was conducted with percentage change from baseline as the outcome variable and HBV and HCV status as the explanatory variable.

The results in Table 7 show that co-infected patients had a large decrease in ALAT (6.9702 IU/L) and a lower percentage of decrease in ASAT (18.5594 IU/L) compared with co-infected HBV/HIV patients while mono-infected patients had a high decrease in albumin (0.8594 mg/dL) and total bilirubin (15.6250 mg/dL) compared with co-infected patients. But HIV/HCV co-infected patients had a bigger decrease in ALAT (16.1733 IU/L), ASAT (23.0274 IU/L), albumin (2.0833 mg/dL) and total bilirubin (18.1818 mg/dL) compared with HIV mono-infected patients.

The independent samples *t*-test in Table 8 shown below indicates the percentage decrease in ALAT was significantly higher in co-infected patients compared with mono-infected patients (*p* = 0.029). No difference was observed when comparing the percentage decrease in ASAT, albumin and total bilirubin between co-infected and non-infected patients.

## 4. Discussion

The guideline for clinical management of HIV patients recommends screening for viral hepatitis; unfortunately this is not standard practice in Cameroon, as it is not included in the package of baseline laboratory tests [9]. Given the limited resources available for population screening efforts, and regular control for some key elements (viral load, CD4 count, ASAT, ALAT, albumin, bilirubin, creatinine), the present study was aimed to evaluate the treatment response of HIV patients and those co-infected with hepatitis B or C. It further sought to identify associated risk factors linked to the treatment outcome among these HIV-1 infected patients placed on HAART, which would contribute toward the understanding of the burden of the viral hepatitis co-infections and at-risk populations in Cameroon.

Out of the 52 HIV patients and 36 HIV/HBV patients, it was seen that the percentage increased in weight over 12 months (17.12 kg) was greater than the percentage increase in CD4 (8.92 cells/mm^3^; see Table 1). However, the percentage decrease in viral load over 12 month period was also very high (1035.17 copies/mL). These positive variations could be due to the close follow-up of patients and good adherence to treatment [20]. The highly significant *p* values (*p* < 0.0001) showed that there was a significant change in mean value from baseline for all three variables. The decrease in viral load and increase in CD4 and weight could be linked to regular therapy and proper follow-up. Adherence to HAART in this population may be directly related to improvements in the CD4 cell count [21].

With respect to variations in weight, we found that mono-infected patients had a much higher increase in weight (mean = 22.57 kg) than the co-infected patients. On the other hand, co-infected patients had a much higher increase in CD4 (mean = 10.41 cells/mm^3^) and a much higher drop in viral load (mean = 1319.67 copies/mL: see Table 2). The differences among the participants could be due to the differences in adherence to treatment and other factors such as alcoholic status, HIV stage at initiation, etc. We however, noted irrespective of infection status, there were positive variations among these variables after 12 months though not significant. This isin line with a study carried out in Bangkok and Thailand that came up with similar results [22]. The increase in CD4 in HIV/HBV patients after 12 months of HAART seem to show that HBV did not adversely affect HAART treatment in HIV patients in our study population [23].

The We equally found a significant difference between creatinine baseline measurement and measurement after 12 months (*p* = 0.020). The significant difference in renal functions between mono-infected and co-infected HIV patients with HBV was due to the percentage change in creatinine from baseline. In this case, co-infected patients have a high increase in creatinine (mean = 11.1111 mg/dL) compared with mono-infected patients (mean = 6.2500 mg/dL). However, the *p* value provided in the independent sample test table (*p* = 0.494) shows that this difference was not statistically significant (see Table 4).

There was a significant decrease in mean ALAT (*p* < 0001) and ASAT (*p* < 0001) values from baseline but with percentage decrease in ASAT over 12 months (20.03), higher than the percentage decrease in ALAT (12.78). Looking at the differences in mean percentage decrease between the ASAT and ALAT, there was alteration of the liver functions which could be linked to HBV or liver damage [24]. There is also a slight decrease in albumin and total bilirubin from baseline but this change is not statistically significant. It was also seen that mono-infected patients have a high decrease in ALAT/ASAT compared with co-infected HBV/HIV patients. Co-infected patients have a high decrease in albumin compared with mono-infected patients.

HIV disease outcomes following first initiation of a HAART regimen was similar in HIV/HCV co-infected patients compared with HIV-only patients in terms of AIDS-free survival and detectable HIV virus during the first 12 months. There was no greater differences in the immune response between the mono-infected compared with the co-infected and this was similar to a cohort study carried in Switzerland which showed an equivalent response in nearly 1600 HCV-positive and HCV-negative patients with respect to their ability to suppress HIV while receiving HAART [16].

The percentage increase in weight over 12 months (17.12 kg) was greater than the percentage increase in CD4 (8.92 cells/mm^3^) at 95% CI. However, the percentage decrease in viral load over a 12-month period was also very high (1035.17 copies/mm^3^). The highly significant *p* values (*p* < 0.0001) showed that there was a significant change in mean value from baseline for all three variables (see Table 2).

It was seen that mono-infected patients have a high increase in weight (mean = 18.65 kg), a slightly high increase in CD4 (mean = 9.20 cells/mm^3^) and a high decrease in viral load (mean = 1111.20 copies/mL) but the percentage increase in weight was not statistically different in co-infection status at *p* = 0.064, percentage increase in CD4 was clearly not different in co-infection status at *p* = 0.544, and percentage decrease in viral load was also clearly not different in co-infection status at *p* = 0.245. This lower increase in CD4 count in co-infected could be due to low immune response to HAART therapy and this was similar to the result of the study carried out on HIV/HCV patients after 12 months of HAART in Australia [23]. This cohort study shows that HIV/HCV patients have a lower CD4 reconstitution, which is consistent with a meta-analysis study carried out by a group of scientists amongst HAART HIV–HCV co-infected patients that also indicated less immune reconstitution, as determined by CD4 cell count after 48 weeks of HAART [25].

There was a significant difference between creatinine baseline measurement and measurement after 12 months (*p* = 0.020). Mono-infected patients had a larger increase in creatinine (mean = 9.0909 mg/dL) compared with patients co-infected with HCV (mean = 0.0000 mg/dL). However, the independent sample test table (*p* = 0.494) showed that this difference was not statistically significant (see Table 4).

The percentage decrease in ASAT over 12 months (20.03) was much higher than the percentage drop in ALAT (12.78) and this was significant [ALAT (*p* < 0001) and ASAT (*p* < 0001)]. Looking at the differences in mean percentage decrease between the ASAT and ALAT, there seemed to be an alteration of the liver function biomarkers which could be linked to HCV or liver damage [24]. There was also a slight decrease in albumin and total bilirubin from baseline but these changes were not statistically significant. (see Table 6). Co-infection with HCV/HIV patients indicated had a larger margin of decrease in ALAT (16.1733 IU/L), ASAT (23.0274 IU/L), albumin (2.0833 mg/dL) and total bilirubin (18.1818 mg/dL) compared with mono-infected patients. Although this result was unexpected given the well-known effects of hepatitis on liver enzymes and the cumulative effect of HAART, the follow-up time might not have been enough to witness biological palpable changes as observed in previous studies.

Our study should be interpreted with caution due to a smaller sample size. We would have ideally done a complete evaluation of hepatitis B infection at baseline among co-infected patients. The follow-up time (12 months) might not have been enough to evaluate all palpable effects identified in this study. However, despite these, our study reports the first attempt to understand effects of hepatitis in a low resource programmatic setting.

## 5. Conclusions

Co-infection with HBV or HCV is relatively common among HIV-infected participants in Kumba Health District. HIV disease outcomes following HAART do not appear to be adversely affected by HBV or HCV co-infection after 12 months, except for slightly poorer CD4 count responses in HIV/HCV co-infected patients. But it is seen that there are differences in response to HAART between the mono-infected compared with the co-infected, taking into consideration the weight, CD4 count, and viral load. Concerning the renal and liver functions, we observed a decrease in all the biomarkers in response to HAART over 12 months, with more rapid decrease in mono-infected patients as compared with co-infected with HBV, but the case was contrary for HCV patients. Future studies are planned to repeat observations after long-term HAART exposure (>5 years).

## Figures and Tables

**Table 1:** Mean immune response measurements and percentage change between 1 and 12 months in both mono- (HIV) and co-infected (HIV and HBV; HIV and HCV) patients.

	Month 1 Mean (SD)	Month 12 Mean (SD)	Percentage Change and 95% Confidence Interval	*p*-Value
**Weight (kg)**	58.92 (16.76)	66.20 (14.16)	17.12 (12.70, 21.55)	<0.0001
**CD4** **(cells/mm^3^)**	510.96 (302.09)	539.08 (306.31)	8.92 (6.44, 11.40)	<0.0001
**Viral load** **(copies/mL)**	65375.08 (197229.84)	4481.37 (12381.16)	1035.17 (684.97, 1385.38)	<0.0001

**Table 2:** Group statistic table for assessment of immune response parameters.

	HBV or HCV Status	N		Mean		Std. Deviation		Std. Error Mean	
		HBV	HCV	HBV	HCV	HBV	HCV	HBV	HCV
**Percentage weight (kg)**	Positive	36	12	7.4348	5.9422	5.20997	4.70470	0.86833	1.35813
	Negative	64	88	22.5788	18.6522	26.11704	23.30631	3.26463	2.48446
**Percentage CD4 (cells/mm^3^)**	Positive	36	12	10.4122	6.8558	17.12586	4.52219	2.85431	1.30544
	Negative	64	88	8.0875	9.2065	9.01223	13.22807	1.12653	1.41012
**Percentage viral load (copies/mL)**	Positive	36	12	1319.6755	477.6668	2650.27716	643.38148	441.71286	185.72824
	Negative	64	88	875.1497	1111.2034	959.53780	1855.72990	119.94222	197.82147

**Table 3:** Mean immune response measurements and percentage change between 1 and 12 months in co-infected and mono-infected patients.

Variables	Categories	1 Month Mean (SD)		12 Months Mean (SD)		Percentage Change and 95% CI		*p*-Values for Change from Baseline		*p*-Values for Difference	
		HBV	HCV	HBV	HCV	HBV	HCV	HBV	HCV	In HBV Status	In HCV Status
**Weight**	**Co-infected with HIV**	62.44 (11.14)	66.25 (10.50)	66.69 (9.62)	69.83 (8.90)	7.43 (5.67, 9.19)	5.94 (2.95, 8.93)	<0.0001	0.001	<0.0001	0.064
	**Mono-infected**	56.94 (19.01)	57.92 (17.24)	65.92 (16.23)	65.70 (14.70)	22.57 (16.05, 29.10)	18.6 (13.71, 23.59)	<0.0001	<0.0001		
**CD4**	**Co-infected**	360.58 (234.45)	406.67 (159.40)	378.33 (231.68)	429.75 (156.10)	10.41 (4.61, 16.20)	6.85 (3.98, 9.72)	0.001	0.026	0.375	0.544
	**Mono-infected**	595.55 (304.44)	525.18 (314.53)	629.50 (307.50)	553.99 (319.08)	8.08 (5.83, 10.33)	9.20 (6.40, 12.00)	<0.0001	<0.0001		
**Viral load**	**Co-infected with HIV**	107520.47 (242869.14)	49912.08 (154947.21)	5213.53 (10022.32)	4394.00 (13248.70)	1319.67 (422.95, 2216.40)	477.66 (68.88, 886.45)	0.005	<0.0001	0.337	0.245
	**Mono-infected**	41668.30 (163622.61)	67483.67 (202958.18)	4069.53 (13587.01)	4493.28 (12338.66)	875.14 (635.46, 1114.83)	1111.20 (718.01, 1504.39)	<0.0001	<0.0001		

**Table 4:** Group statistic table for renal function.

	HBV or HCV Status	N		Mean		Std. Deviation		Std. Error Mean	
		HBV	HCV	HBV	HCV	HBV	HCV	HBV	HCV
**Percentage creatinine (mg/dL)**	**Positive**	36	12	11.1111	0.0000	31.87276	0.00000	5.31213	0.00000
	**Negative**	64	88	6.2500	9.0909	35.07362	35.99605	4.38420	3.83719

**Table 5:** Mean creatinine measurements and percent change between 1 and 12 months in HIV/HBV and HIV/HCV co-infected and mono-infected HIV patients.

Variables	Categories	1 Month Mean (SD)		12 Months Mean (SD)		Percent Change and 95%CI		*p* Values for Change from Baseline		*p* Values for Difference Hepatitis Status	
		HBV	HCV	HBV	HCV	HBV	HCV	HBV	HCV	Between HBV Status	Between HCV Status
**Creatinine level**	**Co-infected**	1.11 (0.31)	1.00 (0.00)	1.00 (0.00)	1.00 (0.00)	11.11 (0.32, 21.89)	0.00 (0.00, 0.00)	0.044	NA	0.020	0.494
	**Mono-infected**	1.14(0.41)	1.14 (0.41)	1.05 (0.23)	1.05 (0.23)	9.09 (1.46, 16.71)	9.09 (1.46, 16.71)	0.020	0.020		

**Table 6:** Mean liver toxicity measurements and percentage change between 1 and 12 months in both HIV mono-infected and HIV/HBV and HIV/HCV co-infected patients.

	Month 1 Mean (SD)		Month 12 Mean (SD)		Percentage Change and 95% Confidence Interval		*p*-Value	
	HBV	HCV	HBV	HCV	HBV	HCV	HBV	HCV
**ALAT (IU/L)**	43.91 (43.93)	43.91 (43.93)	38.56 (38.40)	38.56 (38.40)	12.78 (9.49, 16.06)	12.78 (9.49, 16.06)	<0.0001	<0.0001
**ASAT (IU/L)**	41.64 (46.47)	41.64 (46.47)	34.65 (38.44)	34.65 (38.44)	20.03 (17.09, 22.98)	20.03 (17.09, 22.98)	<0.0001	<0.0001
**Albumin (g/dL)**	4.14 (0.34)	4.14 (0.34)	4.10 (0.30)	4.10 (0.30)	1.10 (−0.22, 2.42)	1.10 (−0.22, 2.42)	0.103	0.103
**Total Bilirubin (g/dL)**	0.92 (0.66)	0.92 (0.66)	0.81 (0.44)	0.81 (0.44)	7.59 (−5.22, 20.41)	7.59 (−5.22, 20.41)	0.242	0.242

**Table 7:** Group statistic table for liver function in HIV/HBV and HIV/HCV co-infected and HIV mono-infected patients.

	HBV Status	N		Mean		Std. Deviation		Std. Error Mean	
		HBV	HCV	HBV	HCV	HBV	HCV	HBV	HCV
**Percentage ALAT**	**Positive**	36	12	6.9702	16.1733	22.78705	4.57881	3.79784	1.32179
	**Negative**	64	88	16.0486	12.3177	10.58259	17.53174	1.32282	1.86889
**Percentage ASAT**	**Positive**	36	12	18.5594	23.0274	13.23796	11.93433	2.20633	3.44514
	**Negative**	64	88	20.8692	19.6300	15.72291	15.21572	1.96536	1.62200
**Percentage albumin**	**Positive**	36	12	1.5278	2.0833	7.91197	7.21688	1.31866	2.08333
	**Negative**	64	88	0.8594	0.9659	5.94650	6.64614	7.4331	7.0848
**Percentage Total bilirubin**	**Positive**	26	11	−21.1538	18.1818	37.87632	40.45199	7.42816	12.19673
	**Negative**	48	68	−15.6250	5.8824	25.59141	59.55645	3.69380	7.22228

**Table 8:** Mean liver function measurements and percentage change between 1 and 12 months in HIV/HBV and HIV/HCV co-infected and HIV mono-infected patients.

Variables	Categories	1 Month Mean (SD)		12 Months Mean (SD)		Percent Change and 95% CI		*p* Values for Change from Baseline		*p* Values for Difference Hepatitis Status	
		HBV	HCV	HBV	HCV	HBV	HCV	HBV	HCV	HBV	HCV
**ALAT**	**Co-infected**	26.63 (19.00)	46.91 (16.61)	24.08 (16.00)	40.50 (14.15)	6.97 (−0.73, 14.68)	16.17 (13.26, 19.08)	0.075	<0.0001	0.029	0.097
	**Mono-infected**	53.62 (50.65)	43.50 (46.47)	46.70 (44.59)	38.29 (40.65)	16.04(13.40,18.69)	12.31 (15.44, 30.61)	<0.0001	<0.0001		
**ASAT**	**Co-infected**	27.05 (12.25)	49.16 (37.00)	22.94 (10.33)	40.91 (33.77)	18.55 (14.08, 23.03)	23.02 (−2.50, 6.66)	0.254	0.339	0.458	0.460
	**Mono-infected**	49.84 (55.86)	40.61 (47.70)	41.23 (46.27)	33.79 (39.13)	20.86 (16.94, 24.79)	19.63 (−8.99, 45.35)	0.713	0.167		
**Albumin**	**Co-infected**	4.13 (0.35)	4.08 (0.28)	4.08 (0.28)	4.00 (0.00)	1.52 (−1.14, 4.20)	2.08 (8.60, 16.03)	<0.0001	<0.0001	0.634	0.590
	**Mono-infected**	4.14 (0.35)	4.14 (0.35)	4.10 (0.31)	4.11 (0.31)	0.85 (−0.62, 2.34)	0.96 (16.40, 22.85)	<0.0001	<0.0001		
**Total bilirubin**	**Co-infected**	0.80 (0.57)	1.08 (0.51)	0.72 (0.45)	0.91 (0.28)	−3.84 (−25.16, 17.46)	18.18 (−0.44, 2.37)	0.252	0.176	0.509	0.512
	**Mono- infected**	0.98 (0.70)	0.89 (0.67)	0.85 (0.43)	0.79 (0.45)	13.20 (−3.04, 29.45)	5.88 (−8.53, 20.29)	0.109	0.418		

## Data Availability

The data presented in this study are available in the manuscript.
